# Impacts of the Covid‐19 pandemic on the health of university students

**DOI:** 10.1002/hpm.3145

**Published:** 2021-03-11

**Authors:** Laura Ihm, Han Zhang, Alexandra van Vijfeijken, Mark G. Waugh

**Affiliations:** ^1^ Division of Medicine UCL Royal Free Campus University College London London UK

**Keywords:** coronavirus, Covid‐19, health, pandemic, university students

## Abstract

The Covid‐19 pandemic caused by the novel Sars‐CoV‐2 coronavirus, has resulted in millions of deaths and disruption to daily life across the globe. University students have been additionally affected by a sudden move to online learning, the closure of campuses and dramatic societal changes that have upended their experiences of higher education. Here we focus on the physical and mental health consequences of the pandemic for this population sector during 2020, and the interdependencies of these impacts. We survey the challenges for infection control on campuses and for monitoring the disease dynamics in student communities. Finally, we explore the psychological and mental health problems that have been exacerbated by the pandemic and evaluate the underlying factors that are most relevant to students.

## 1 INTRODUCTION

The first outbreak of Covid‐19 caused by the novel Sars‐Cov‐2 coronavirus was reported in December 2019 in Wuhan, China.^1^ The disease rapidly spread in 2020 and the ensuing global Covid‐19 pandemic^2^ has resulted in large‐scale loss of life, debilitating illness and major socioeconomic disruption.^3^ The disease has severely disrupted education at all levels and consequently, the lives of students of all ages.^4^, ^5^ Throughout the world, university students have been affected through campus closures, unplanned rapid shifts to online learning and the introduction of non‐pharmaceutical interventions (NPIs) such as social distancing, mask wearing and travel restrictions to curb transmission of the virus.^6^, ^7^ In the United Kingdom (UK), the first confirmed cases of Covid‐19 were an international student from the University of York and his mother who was visiting from China on 31st January 2020. The first recorded death from the pandemic in the UK was on 5th March 2020. On 23rd March 2020—as case numbers rapidly escalated, a national ‘stay at home’ lockdown was announced, which led to the immediate suspension of face‐to‐face teaching and the closure of university facilities. These massive changes have had ramifications for the educational experiences of university students and their mental, physical and financial well‐being.

In a world consumed by the battle with Covid‐19, 2020 was also a time of civil unrest and political uncertainty. There were numerous street demonstrations triggered by public outrage at the fatal shooting of George Floyd on the 25th of May 2020 in Minneapolis. Some Asian students suffered racial abuse motivated by the epidemic's likely origins in China.^8^ ‘Panic‐shopping’ led to temporary shortages of essential food and hygiene products. Misguided anti‐vaccination and lockdown sceptics staged protests and disseminated misleading conspiracy theories via social media seeking to undermine official efforts to combat the pandemic. This unsettling atmosphere was augmented by a bitterly divisive US Presidential election campaign where academic scientists and their public health advice were frequently in the spotlight and criticized. In the UK, protracted Brexit negotiations have had implications for research funding, access to the Erasmus student exchange scheme and the future of European Union (EU) students studying in the UK. Despite this turbulent backdrop, the basics of university life managed to grind on. Exams still happened and students still graduated. Teaching even continued; but more often as pre‐recorded or online lectures as opposed to the traditional face‐to‐face format. Nevertheless, 2020 was for many students an annus miserabilis—an extended period of depressing news, and for some, isolation, illness, bereavement, or financial hardship.

There is now a large body of published, peer‐reviewed research papers assessing the impacts of the pandemic on university students, the majority of these are surveys investigating topics such as student adaptations to online learning,^9^, ^10^, ^11^, ^12^, ^13^, ^14^, ^15^, ^16^ Covid‐19 impacts on mental health,^4^, ^5^, ^17^, ^18^, ^19^, ^20^, ^21^, ^22^, ^23^, ^24^, ^25^, ^26^ or less frequently, both of these factors in parallel.^27^, ^28^, ^29^, ^30^ Other studies that have taken a broader approach to understand how the pandemic has altered academic, societal, health, lifestyle and behavioural elements of the student experience.^31^, ^32^, ^33^, ^34^, ^35^ In this commentary we seek to focus more on the medical impacts, and particularly the enormous practical public health challenges posed by the Covid‐19 pandemic for university students.

Our perspectives of the pandemic have been influenced by our experiences as students and staff of the Integrated Medical Sciences (IMS) undergraduate degree programmes at University College London (UCL), situated in London, England; a city that has suffered high per capita excess mortality^36^ and has undergone radical societal change during the Covid‐pandemic.^3^, ^6^ In this commentary we chart how Covid‐19 has affected university students from a public health perspective and to identify lessons that may inform future practice in this ‘era of pandemics’

### 1.1 The pandemic on campus—physical health impacts & epidemiology

When universities shut their doors and students emptied out of their campus accommodation in March 2020 the degree to which younger people were susceptible to falling ill with Covid‐19 was incompletely understood. The earliest outbreaks in China and Northern Italy indicated that the risk of being hospitalised or dying from Covid‐19 increased proportionally with age and the presence of pre‐existing comorbidities such as diabetes or hypertension.^37^ It was becoming apparent at that point that young adults generally suffered milder symptoms and furthermore, a substantial number could be asymptomatic but still infectious. An in‐depth multidisciplinary investigation mapping the transmission pattern of Covid‐19 in a group of US college students during the 2020 Spring Break concluded that 21.9% of infected individuals were asymptomatic^38^ and the rest suffered from the already established mild known symptoms of Covid‐19 such as headaches, anosmia, sore throat, coughing, shortness of breath, diarrhoea, fatigue and less commonly, fever. Interestingly, a similar number of students in the affected cohort also suffered from these symptoms but were subsequently found by RT‐PCR not to be infected with Sars‐CoV‐2, which underscored at this early stage the importance of sensitive diagnostic tests to differentiate Covid‐19 from other circulating viruses such as influenza. It is relevant to note that physical illness such as seasonal respiratory infections are associated with poor learning outcomes^39^ and the common presenting symptoms of mild cases of Sars‐CoV‐2 infection are likely to be detrimental for learning. The finding that Covid‐19 has a less severe presentation in typical university age patients has not altered over the course of 2020. There has been one notable exception though; an investigation into the effects of Covid‐19 on fit young college athletes reported that Sars‐CoV‐2 infection could be associated with longer term cardiac abnormalities,^40^ but this has not yet been firmly established as a general consequence of infection with the virus.^41^

In England, substantial falls in case numbers over the summer of 2020 prompted the gradual easing of lockdown restrictions. During August 2020, the reopening of most sectors of the economy and a resumption of international travel, engendered a sense that the worst was over, and that normality was slowly re‐emerging from the shadow of Covid‐19. Unfortunately, such guarded optimism turned out to be ill‐founded. In some respects, the scene was being set for another public health crisis. After students flooded back onto campus during September and October 2020 there were several large‐scale outbreaks of Covid‐19 in university settings. It is noteworthy that this development had been largely foreseen. The UK government's own expert scientific advisory group for emergencies (SAGE) and independent authorities in public health and epidemiology had counselled that large population movements and the subsequent concentration of students from different geographical regions would pose a risk for increasing viral transmission.^42^ Moreover, clusters of Covid‐19 cases in the United States where the semester started earlier, had drawn attention to the high‐density, communal living arrangements on campuses which were conducive to the spread of the disease.^43^, ^44^

Aside from financial imperatives, the return to university in England was mainly well intentioned. Laudable aims included restoring face‐to‐face instruction and some level of social interaction. To mitigate infection risks, institutions adopted a range of measures to facilitate the recommencement of in‐person activities. International students were required to quarantine for 14 days upon arrival in the country, although similar restrictions were not imposed on UK‐domiciled students. There were also policies to rigorously implement NPIs to create a so‐called ‘Covid‐secure’ environment. Subsequent investigations into campus outbreaks at the Universities of Exeter and Loughborough indicated that they were mainly due to transmission of the virus in shared student accommodation as opposed to classroom settings.^45^ This difference may be explained by the implementation of additional NPIs such as the mandatory wearing of face masks and limits to room occupancy in teaching environments.^43^ While hospitalisations and deaths remained rare in the main 18–24‐year old student age group, infection control measures led to many having to self‐isolate, alone, in their bedrooms, sometimes with limited or unreliable access to food, and this provoked widespread concerns for their welfare. In line with public health guidelines, these quarantine measures were also extended to identified close contacts of diagnosed students which precipitated a domino effect resulting in whole corridors, and even entire residential buildings being placed under lockdown. There was also a knock‐on effect for relationships with local communities. Co‐incident surges in the recorded number of coronavirus cases in cities with large student populations such as Manchester and Nottingham, ultimately resulted in these regions being placed under tighter restrictions by the government. Although it Is not proven that the increases in Covid‐19 cases in adjacent areas were initiated by infected students, there is now more convincing evidence that increased attack rates in the 18–24‐year old age category did lead to a resurgence of the pandemic more generally in the UK between September and November 2020.^46^ Hence, although serious illness did not commonly feature in the Covid‐19 outbreaks that occurred on campuses at the beginning of the academic year, there was nevertheless serious and prolonged disruption to the student experience. Part of the rationale for reopening campuses was to facilitate limited social activities for a more enriching student experience, however, the numerous Covid‐19 outbreaks in residential settings ended up achieving the opposite in many instances.

Did student behaviour and poor adherence to the rules drive the resurgence of Covid19 that occurred throughout the autumn of 2020? The available evidence suggests that this was not the case and instead demonstrates that students were well‐informed and complied with the restrictions introduced to curb the spread of Covid‐19.^47^, ^48^ A national Student Covid Insights Survey undertaken in the last 3 months of 2020 found that 90% of students have followed the rules and this was a similar compliance rate to that self‐reported by the general population.^49^ These findings paint a more positive picture of students as law‐biding citizens and challenge negative portrayals of their attitudes to NPIs, despite extensive scepticism about governmental policies and behaviour.^50^

In hindsight, the absence of a test, trace and isolate system to identify infectious students at the beginning of the academic year^51^ may have contributed to the surge in Covid‐19 numbers in the first few weeks of the 2020–2021 academic year. To avoid the reverse scenario of students introducing Covid‐19 from universities to their home environments over the Christmas holidays, the UK government recommended that students stagger their departures at the end of term 1 and introduced on‐campus lateral flow antigen‐based testing (LFT) to detect asymptomatic infections. This scheme was subsequently expanded into a national mass asymptomatic testing programme for educational settings overseen by the National Health Service test and trace organisation. However, there is experimental evidence that LFTs are less sensitive at detecting Sars‐CoV‐2 when compared to the gold‐standard RT‐PCR tests^52^ prompting concerns that false negative tests could seed subsequent outbreaks of Covid‐19.^53^, ^54^ Despite these misgivings, LFTs have practical advantages in that they can deliver results within two hours of the sample being taken. Furthermore, LFTs are amenable to high throughput and more frequent testing, which renders this method more feasible for large‐scale community‐level surveillance of infection levels, especially when conducted on a twice weekly basis with the availability of follow‐up RT‐PCR confirmation when a positive‐test is detected. Interestingly, LFTs carried out at end of term during the first 2 weeks of December 2020 at different universities reported exceedingly low levels of asymptomatic infection amongst students,^55^ with some universities not detecting any positive cases at all.^56^ These are surprising findings given that the nationwide rate of infection for the 12–24 years demographic was estimated as being between 1.0% and 1.5% during early December 2020.^57^ A coordinated track, test and isolate scheme is an essential facet of an information‐led, public health strategy for Covid‐19 disease control,^58^, ^59^ and it will be interesting to see if the LFT programme will be effective in crowded university environments when these institutions eventually repopulate more fully.

One of the major problems in assessing the prevalence and dynamics of the Covid‐19 pandemic in UK universities has been the paucity of reliable student‐specific data, and particularly so at the beginning of semester when thousands of students converged on campuses without any prior screening. Most of the publicly available data in this domain has not been collected or collated in a standardised format which makes it difficult to make cross comparisons or draw firm conclusions.^60^ As an example, official data collected for the 17 to 24 age category includes high school students, people who are not in higher education and those who have already graduated. Furthermore, most studies do not record ‘student’ as an occupation or single out student accommodation when asking about household size. As regards the information that is available at the time of writing (January 2021), figures compiled by the University and College's Union have recorded over 56,000 confirmed cases of Covid‐19 in UK higher education institutes, which typically corresponds to between 3% and 9% of their combined staff and student populations.^61^ These case numbers should be treated as an estimate, as the numbers of people routinely present on campus during this timeframe would be fewer than in previous years due to most staff and students either working or studying from home. At a national level, anti‐Sars‐CoV‐2 antibodies— indicating a recent infection with the virus, were detectable in approximately one eighth of the UK population in December 2020.^62^ These different data sets, although not directly comparable, may suggest that levels of Covid‐19 in university settings did not exceed those in the general population but nevertheless, the level of disruption to university life has still been unprecedented in peacetime.

### 1.2 Covid‐19 impacts on student mental health

The mental health of the student population has been a major concern for several years and the pandemic has certainly exacerbated this problem. Available data from the UK has consistently shown that just over half of university student have experienced worsened mental health in 2020 (reviewed in^49^). Self‐reported deteriorations in student mental health, presenting as augmented psychological stress, anxiety, depression and even post‐traumatic stress disorder,^20^, ^63^ appears to be a global phenomenon.^5^, ^27^, ^35^, ^64^, ^65^, ^66^, ^67^, ^68^, ^69^, ^70^, ^71^, ^72^ In common with the general population, isolation, loneliness and worries about catching the disease have been found to be drivers of poor psychological health.^5^, ^73^ Specifically for students, the emergency transition to online learning has had mixed satisfaction outcomes and has found to be a stressor, particularly when associated with increased workloads and where learning technologies or internet connectivity are inadequate.^27^, ^33^, ^74^, ^75^, ^76^ The vulnerability of family members to Covid‐19 infection has also emerged as a source of worry for many students.^73^ Familial support networks represent an important source of psychological support for students^67^, ^70^ and bereavement or serious illness affecting relatives, or indeed the fear of such events happening^77^ have been a significant cause of poor mental health in 2020.^66^ However, this is not always a straightforward positive relationship and at least one study has found that for a small proportion of students, increased family conflicts during lockdown have negatively affected their mental well‐being.^78^

It is interesting to note as well that the pandemic has positively affected the experiences of some university students and there is some information available on the coping mechanisms that they have benefited from.^79^, ^80^, ^81^, ^82^ To date, pre‐existing high levels of self‐efficacy and physical exercise have emerged as positive determinants of student mental health resilience.^19^, ^66^, ^83^, ^84^, ^85^ There is even evidence that student performance in examinations may have improved during lockdown and this further demonstrates the heterogeneous impacts of the pandemic on the student experience.^86^ It may be some time though before we fully appreciate the long‐term ramifications of the Covid‐19 pandemic for student mental health as it is now becoming established that psychiatric complications are a feature of Long‐Covid; a chronic syndrome where 10%–20% patients do not fully recover from Covid‐19 even several months after their initial infection.^87^

To fund their living costs, university students often rely on casual employment in the industry sectors that have been most affected by NPIs such as in cafes and restaurants, or as assistants in non‐essential retail. Comprehensive analyses from a major study in the United States conducted during the early days of the crisis in April 2020 uncovered a clear correlation between Covid‐19 financial shocks and negative impacts on the academic progress of poorer students.^31^ Similarly, a cross sectional survey from Bangladesh found that the financial hardship caused by the pandemic was associated with student anxiety and depression.^88^ This association has also been noted amongst the general population in India during the early stages of the pandemic.^89^ An association between socioeconomic deprivation and poor educational outcomes is well establish. In the UK there have been some attempts to address this issue with for example, modest increases in governmental contributions to student hardship funds, but the extent and duration of such financial support may need to be reconsidered if the economic damage caused by the pandemic is sustained.

## 2 CONCLUSIONS

The advent of efficacious vaccines may herald a gradual return to campus normality during 2021, but the pace of improvement will depend on factors such as vaccine availability and the prioritisation status of university teachers and learners in national vaccination schemes. As travel bans remain in place, international conferences, student exchange schemes and placements remain problematical or impossible. Even in countries where mass vaccinations are progressing satisfactorily, concerns about more transmissible or lethal Sars‐CoV‐2 variants may mean that many facets of academic life will likely remain stunted for some time. The events of 2020 have shown that university campuses, and particularly student living arrangements, pose substantial challenges for controlling Sars‐CoV‐2 dissemination and that NPIs on their own are insufficient to fully suppress Covid‐19 in such environments. In addition to NPIs, experience has highlighted the importance of systematic and concerted testing programmes to monitor community transmission and the need to have schemes in place to ensure the welfare of students who are required to quarantine. Finally, policy priorities and resource allocation may need to be rethought to provide additional targeted assistance to students who have been impacted by Covid‐19‐associated psychological trauma, bereavement, or financial instability.

## CONFLICTS OF INTEREST

The authors declare no conflicts of interest.
